# Dopamine-functionalized mesoporous onion-like silica as a new matrix for immobilization of lipase *Candida* sp. 99-125

**DOI:** 10.1038/srep40395

**Published:** 2017-01-09

**Authors:** Junkai Gao, Yanjun Jiang, Jinshu Lu, Zhi Han, Jiajia Deng, Yan Chen

**Affiliations:** 1 School of Maritime and Civil Engineering, Zhejiang Ocean University, Zhoushan, 316022, China; 2 School of Chemical Engineering and Technology, Hebei University of Technology, Tianjin, 300130, China

## Abstract

Dopmine functionalized mesoporous onion-like silica (DPMS) was synthesized via a biomimetic coating, and lipase *Candida* sp. 99-125 (LCS) was immobilized in DPMS (LCS@DPMS) by physical adsorption in this study. The DPMS was characterized by SEM, TEM, BET and FT-IR, and it was shown that the DPMS had clear multishell structures with large surface area of 419 m^2^/g. The activity, pH stability, thermal stability, storage stability, and reusability of the LCS@DPMS were investigated in detail. The stabilities of LCS@DPMS were improved significantly compared to the free lipase and LCS@MS (LCS immobilized in unfunctionalized mesoporous onion-like silica by physical adsorption). All the results indicated that the DPMS had high efficiency and improved stability for lipase immobilization.

Enzyme-catalyzed processes are widely used in the pharmaceutical or food industry due to their ease of production, green chemistry and substrate specificity^1^. The use of free enzymes is often hampered by several drawbacks such as low stability, high cost and poor reusability^2^. Immobilization of enzymes on solid supports is one of the best methods for biocatalysis to increase the stability and reusability of enzymes, simplify purification and recycling processes^3^,^4^. In the last few decades, numerous immobilization approaches and supports materials have been investigated^5^. In particular, mesoporous silica supports have attracted much attention due to their well-defined pore sizes and geometry, large specific surface area, and mechanical stability^6^,^7^. These advantages of mesoporous silica supports enable the improvement of activity and stability of enzymes. Recently, a novel meso-structured onion-like silica (Meso-Onion-S), which had highly curved mesopores and highly ordered onion-like multilayer, was synthesized and used as a host for the immobilization of enzyme, and the stability of the enzyme was effectively improved^8^,^9^. However, there are only two reports regarding the study of Meso-Onion-S, and therefore further studies for the immobilization of enzyme in this novel porous material are indeed required.

Typical strategies for immobilizing enzymes onto mesoporous silicas rely on surface grafting via polymers containing functional groups or low molecular weight linkers to which enzymes are reacted by covalent conjugation^10^,^11^,^12^. But such immobilization methods are usually accompanied by enzymes deactivation^13^. Thus, despite numerous reported approaches for immobilizing enzymes on mesoporous silicas, there is still the need for a simple, facile and cost-effective surface modification approach. Recently, inspired by the adhesive protein secreted by mussels, a mild approach of surface modification utilizing dopamine as functionalization reagent which can be simply attached onto almost all types of material surfaces, has been reported by Messersmith and co-workers^14^. Furthermore, the dopamine molecules contain both catechol and primary amine, which are capable of immobilizing biomolecules^15^,^16^. Therefore, immobilizing enzymes onto different substrates modified with dopamine can be easily achieved by facile conjugation of proteins^12^,^15^,^16^,^17^. However, to the best of our knowledge, Meso-Onion-S modified with dopamine has never been used as enzyme immobilization support. Ongoing efforts are necessary for the study on immobilization of enzyme in this new matrix.

Lipase is widely used in the food, fine chemical and pharmaceutical industries because of the versatile reactions it catalyses, such as hydrolysis, transesterification and esterification^18^,^19^,^20^,^21^. Lipase from *Candida* sp. 99-125 is an important enzyme with high activity and stability, and it is also cost-effective compared to other lipases^22^. Therefore, lipase *Candida* sp. 99-125 has great potential for applications in industry. However, to the best of our knowledge, there is no report for the immobilization of lipase *Candida* sp. 99-125 onto supports modified with dopamine so far. Thus, in this work, Meso-Onion-S was functionalized with dopamine (DPMS) via a biomimetic coating, and lipase *Candida* sp. 99-125 (LCS) was immobilized in DPMS (LCS@DPMS) by physical adsorption. This is the first time to explore the feasibility of immobilizing enzyme in Meso-Onion-S via a biomimetic coating. The activity, pH stability, thermal stability, storage stability, and reusability of the LCS@DPMS were investigated in detail. The results demonstrated that the DPMS exhibited high efficiency and improved stability for lipase immobilization, and it had great potential for practical applications.

## Materials and methods

### Materials

Poly (ethylene glycol)-b-poly (propylene glycol)-b-poly (ethylene glycol) (P123), tetraethoxysilane (TEOS) and dopamine·HCl were purchased from Sigma-Aldrich. 1,3,5-trimethylbenzene (TMB) was purchased from Meryer, China. Lipase *Candida* sp. 99-125 (the protein concentration in the crude lipase is 104.7 mg/g) was purchased from Beijing CTA New Century Biotechnology Co., Ltd., China. All other reagents were of AR grade.

### Preparation of dopamine-functionalized Meso-Onion-S (DPMS)

The Meso-Onion-S was synthesized according to the method reported by Jun *et al*.^8^. Meso-Onion-S was functionalized with dopamine using the post-grafting method. Typically, 0.5 g of Meso-Onion-S was added into 100 mL of 1.25 g/L dopamine solution, which was freshly prepared in Tris-HCl buffer (pH 8.5), and the suspension was stirred for 2 h. Then, the solid was filtered and washed with phosphate buffer (pH 8.0) for three times. The as-prepared solid product was denoted as DPMS.

### Immobilization of lipase in DPMS

Immobilization of lipase in DPMS was performed by physical adsorption method. 50 mg of DPMS was mixed with 5 mL of lipase solution with different concentrations (0.1 M phosphate buffer, pH 8.0) and the suspension was stirred with 200 rpm at 25 °C for 30 min. Then the mixture was centrifuged, washed with phosphate buffer (0.1 M, pH 8.0) for three times, freeze dried in a vacuum chamber, and stored in a fridge under −20 °C. The immobilized lipase was labelled as LCS@DPMS. The loading amount of lipase in DPMS was determined by the Bradford protein assay using bovine serum albumin as a standard. Furthermore, lipase *Candida* sp. 99-125 was also immobilized in Meso-Onion-S by physical adsorption (named LCS@MS) to compare it with that of LCS@DPMS.

### Enzymatic activity assay

Enzymatic activities of native or immobilized lipase were assayed by the hydrolysis of 5 mg/mL 4-nitrophenyl palmitate (pNPP) in ethanol. Typically, 200 μL of pNPP was added into the enzyme solution (0.1 M phosphate buffer, pH 7.4), and the mixture was shaken at room temperature for 3 min^20^. After the reaction, the suspension was filtered and the concentration of the reaction product of p-nitrophenol (pNP) was measured at 405 nm. One unit of lipase activity was defined as that the quantity of enzyme which under the assay conditions liberates 1 μM of pNP per minute.

### Determination of pH optima

In order to determine the optimum pH value, the free and immobilized lipase were incubated in 0.1 M phosphate buffer solutions with different pH values (5.0, 6.0, 7.0, 8.0 and 9.0) at 25 °C for 4 h, and the residual activities were measured. The excessive acidity and alkalinity of the phosphate buffer solutions were adjusted by 0.1 M HCl or 0.1 M NaOH. The relative activity was defined as the percentage of the residual activity compared to the initial activity.

### Thermal and storage stability

The thermal stability was determined by immersing the free and immobilized lipase in isooctane at 60 °C and incubated for a certain period of time, and the residual activities were measured at each time point. The relative activities were calculated as described above.

To determine the storage stability, the free and immobilized lipase were immersed in 0.1 M phosphate buffer solutions with pH 8.0, and stored in a fridge under 4 °C. The residual activities were measured at each time point, and the relative activities were calculated.

### Stability of free and immobilized lipase in shaking conditions

The free lipase, LCS@MS and LCS@DPMS were immersed in 0.1 M phosphate buffer solutions with pH 8.0 at room temperature and shaken at a speed of 200 rev/min. The residual activities were measured at each time point, and the relative activities were calculated.

### Determination of kinetic parameters

The Michaelis-Menten kinetic parameters of free and immobilized lipase were determined by measuring the lipase activity using p-NPP as substrate in the initial concentration range from 1.32 to 13.2 μmol/L at 298 K and pH 8.0. The values of kinetic parameters K_m_ and V_m_ were obtained by fitting the experimental data to the Michaelis-Menten model using non-linear regression code.

### Reusability

The reusability of immobilized lipase was determined by conducting the enzymatic activity assay method described in activity assay section. At the end of each batch reaction, the immobilized lipase was recovered with centrifugation, washed excessively with buffer solution in order to remove the product or substrate and dried at room temperature. Then, the lipase was applied again in the next reaction cycle with fresh substrates.

### Characterizations

SEM images of DPMS were obtained by a scanning electron microscopy (S-4800, Hitachi, Japan). TEM images were realized by a transmission electron microscopy (JEM-2100F, JEOL, Japan) operating at 200 kV. The surface-area measurements of Meso-Onion-S and DPMS were based on the Brunauer-Emmett-Teller method (BET, BELSORP-max, BEL, Japan), and the pore size distributions were obtained from adsorption isotherm branches by the Barrett-Joyner-Halenda (BJH) method. The Fourier transform infrared (FT-IR, VECTOR22, Bruker, Germany) spectra of Meso-Onion-S, DPMS and LCS@DPMS were recorded using the KBr pellet method.

## Results and Discussion

### Characterization of the immobilized lipase

**Figure 1:**
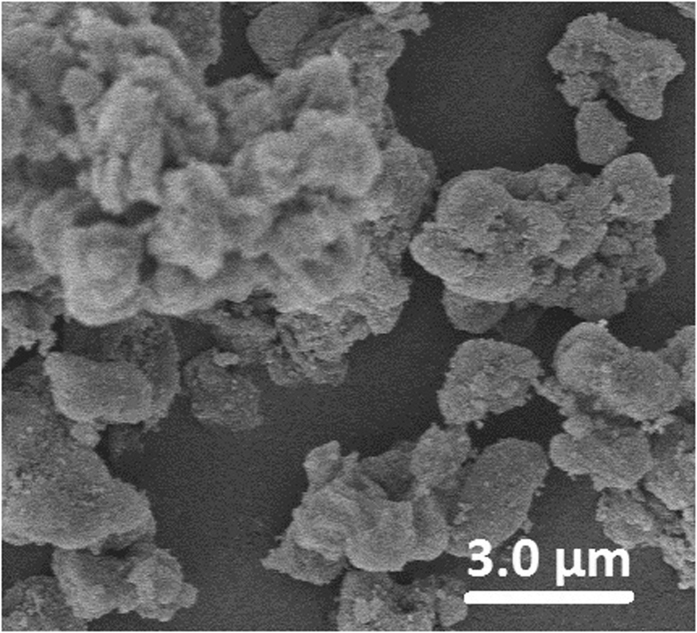
SEM photograph of DPMS.

**Figure 2:**
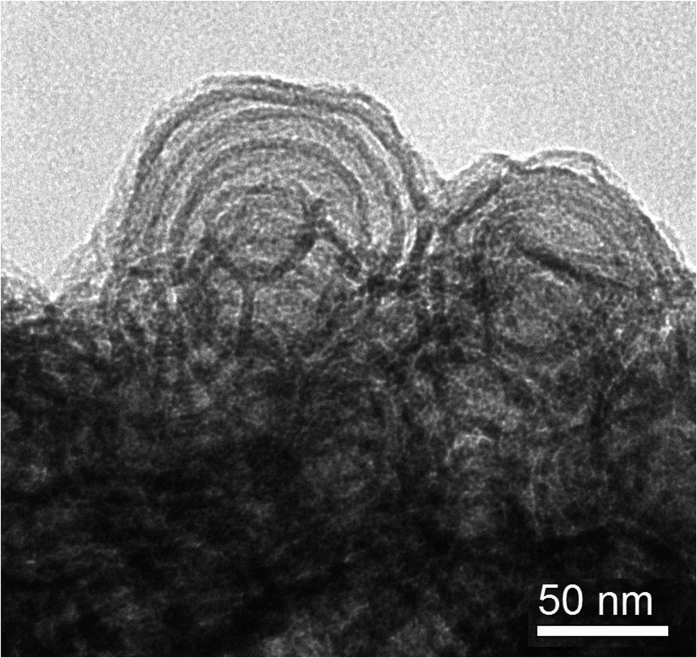
TEM image of DPMS.

The SEM photograph revealed that the DPMS was made up of hundreds of nanometer sized onions which aggregated into secondary micrometer sized particles (Fig. 1), and this result was in accordance with the previous reports^8^,^9^. The TEM image showed that the DPMS had clear multishell structures with mesopore size of about 13 nm between the layers (Fig. 2).

**Figure 3:**
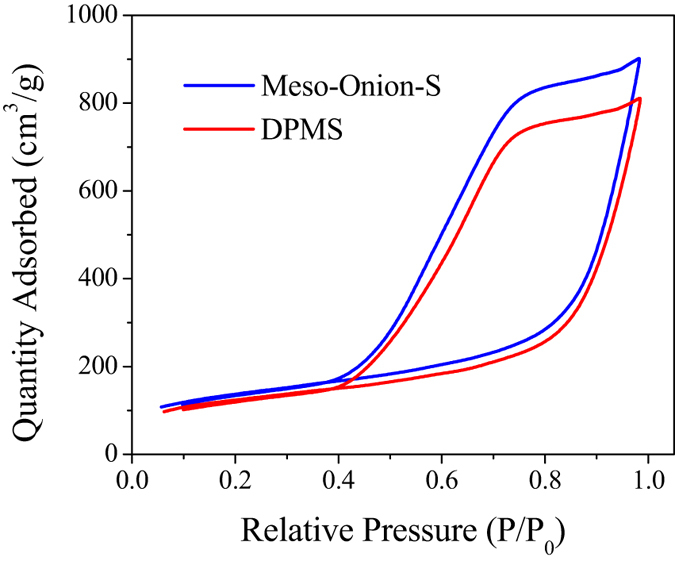
N_2_ adsorption/desorption isotherms of Meso-Onion-S and DPMS.

Figure 3 shows the nitrogen adsorption/desorption isotherms of Meso-Onion-S and DPMS. The specific surface area of DPMS was estimated as 419 m^2^/g, and the BJH adsorption cumulative volume and pore size were 1.14 cm^3^/g and 12.3 nm, respectively. Such pore size and pore volume were large enough to ensure the accommodation of enzymes. Compared with unfunctionalized Meso-Onion-S, the reduction of specific surface area, cumulative volume and pore size of DPMS indicated the success of modification.

**Figure 4:**
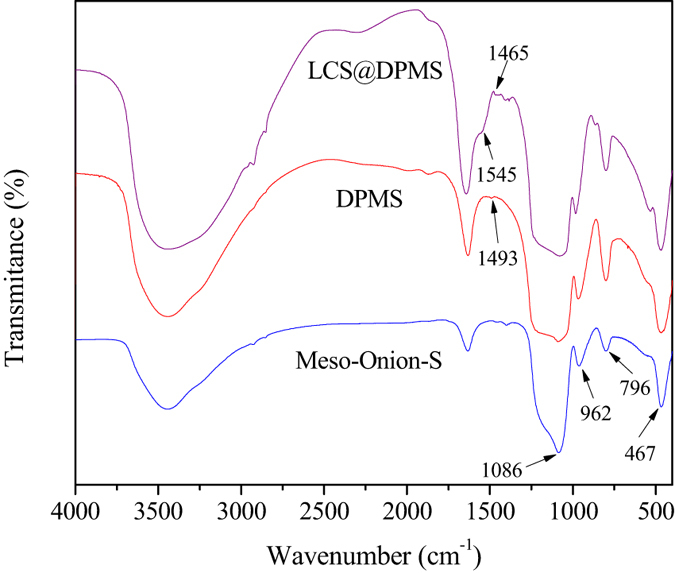
FT-IR spectra of the Meso-Onion-S, DPMS and LCS@DPMS.

Figure 4 exhibits the FTIR spectra of Meso-Onion-S, DPMS and LCS@DPMS. Typical peaks at 467 cm^−1^, 796 cm^−1^ and 1086 cm^−1^ corresponded to the bending vibration, symmetric stretching and asymmetric stretching of Si-O-Si bonds from all of the samples, suggesting that the structure of Meso-Onion-S was well preserved in DPMS and LCS@DPMS^23^. Characteristic band at 962 cm^−1^ represented the symmetric stretching vibration of Si-OH groups^24^. The adsorption peak at 1495 cm^−1^ of DPMS was belong to the stretching of benzene ring in the polydopamine^17^, which indicated the successful immobilization of dopamine. For the LCS@DPMS, the peaks at 1545 cm^−1^ and 1465 cm^−1^ could be assigned to the deformation vibration of -NH_2_ groups and bending vibration of the -C-H groups^2^. This observation confirmed the successful immobilization of the lipase in DPMS.

**Figure 5:**
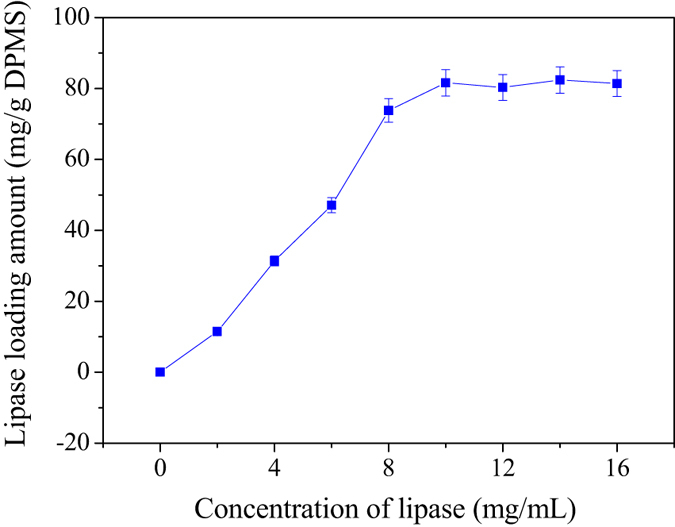
Effect of lipase concentration on the lipase loading amount of LCS@DPMS.

**Figure 6:**
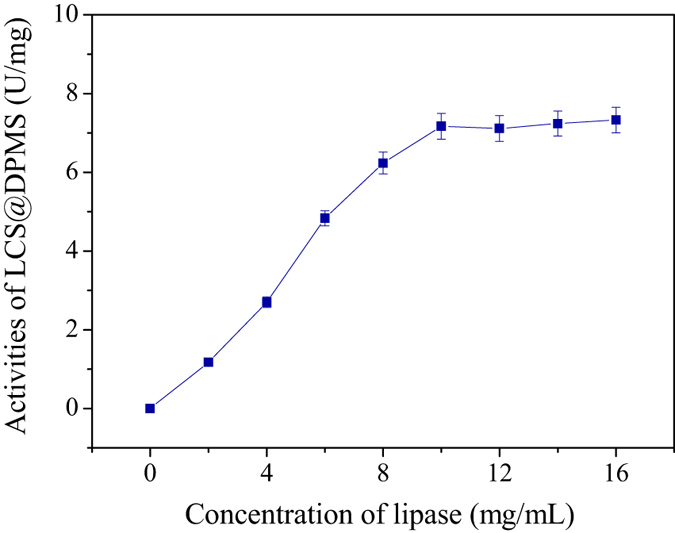
Effect of lipase concentration on the activities of LCS@DPMS.

Figures 5 and 6 shows the effect of lipase concentration on the lipase loading amount and activities of LCS@DPMS, respectively. According to the results, the lipase concentration was set at 10 mg/mL in the subsequent enzyme immobilization experiments. The results indicated that the lipase loading amount of LCS@DPMS was 81.6 mg/g carrier, and the immobilized yield was 52.4%. In the control experiment, the lipase was also immobilized in Meso-Onion-S by physical adsorption (named LCS@MS) under the same preparation conditions. However, the enzyme loading amount of LCS@MS was 62.2 mg/g carrier, and the immobilized yield was 45.3%, which was lower than that of LCS@DPMS. The improved lipase loading capacity and specific activity of LCS@DPMS could be attributed to the strong conjugation of covalent bond between the lipase molecules and the dopamine functionalized Meso-Onion-S, which might promote the lipase loading amount of LCS@DPMS.

### Effect of pH on the activities of free and immobilized lipase

**Figure 7:**
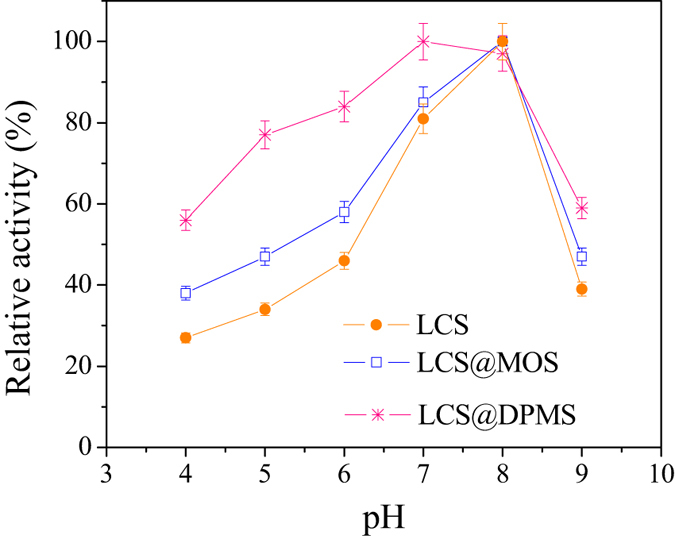
Effect of pH on the activities of free and immobilized lipase. (The 100% relative activity was 13.7 U/mg, 6.30 U/mg, and 7.48 U/mg for free lipase, LCS@MS, and LCS@DPMS, respectively).

The effect of pH on the activities of free and immobilized lipase is shown in Fig. 7. The optimum pH for free lipase and LCS@MS was observed at 8.0, while LCS@DPMS reached its maximal activity at 7.0. Compared to the free lipase and LCS@MS, the optimum pH of LCS@DPMS exhibited a shift toward the acidic region. The polydopamine on the surface of DPMS contains quinones, which are reactive toward nucleophilic groups and can react with the amino or thiol groups of the lipase, and this reaction can lead to the immobilization of lipase in DPMS via covalent bond formation between the polydopamine and the lipase molecules^16^,^25^. The more acidic optimal pH of LCS@DPMS might be caused by the following two reasons: (i) the covalent attachment between the lipase and polydopamine on the surface of the support that limited the transition of lipase conformation against the change of pH^12^,^26^, and (ii) the amine or imines groups on the surface of polydopamine near the active sites of lipase molecules could diminish the concentration of protons^15^,^17^. Additionally, the pH profile of LCS@DPMS was wider range than free lipase and LCS@MS, which enhanced stability of LCS@DPMS under acidic conditions. Therefore, the LCS@DPMS was more suitable for widespread practical applications.

### The thermal stability of free and immobilized lipase

**Figure 8:**
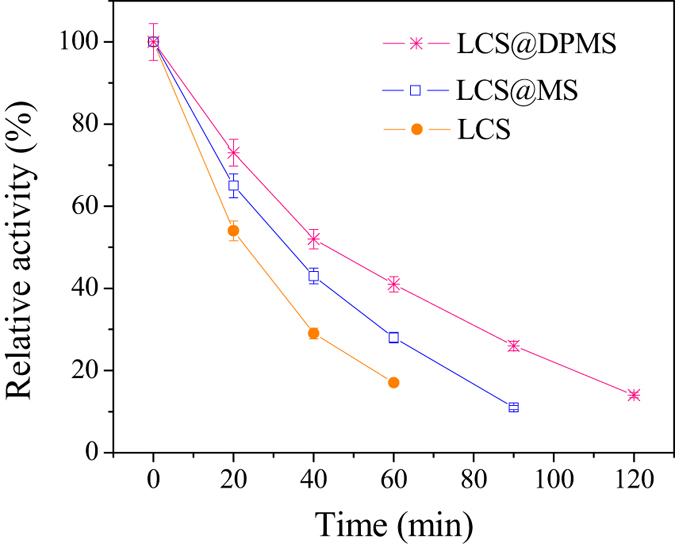
The thermal stability of free and immobilized lipase. (The initial activity was 13.7 U/mg, 6.30 U/mg, and 7.26 U/mg for free lipase, LCS@MS, and LCS@DPMS, respectively).

Thermal stability is an important property of enzyme for industrial applications. The results of thermal stabilities of free lipase, LCS@MS and LCS@DPMS are shown in Fig. 8. Obviously, after 20 min of incubation at 60 °C, the free lipase and LCS@MS retained 54% and 65% of their original activities, respectively, while the remaining activity of LCS@DPMS was 73%. After 60 min the remaining activities of free lipase and LCS@MS decreased to 17% and 28%, respectively, and that of LCS@DPMS decreased to 41%. The half-life of lipase *Candida* sp. 99-125 at 60 °C was prolonged 3.31 times from 12.8 min (free lipase) to 42.4 min (LCS@DPMS). The multipoint interactions between the lipase and the polydopamine on the surface of DPMS could promoted the exposure of the catalytic domain of lipase molecules through conformational changes to the open lid domain, and then enhanced the adsorption of substrate molecules to the active sites^27^,^28^,^29^,^30^. Moreover, the covalent bonds between the lipase molecules and polydopamine could enhance the rigidity of lipase, and protected them from unfolding and then prevented their denaturation at high temperature^31^,^32^,^33^. For these reasons, the thermal stability of LCS@DPMS was meliorated comparing with the free lipase and LCS@MS.

### The storage stability of free and immobilized lipase

**Figure 9:**
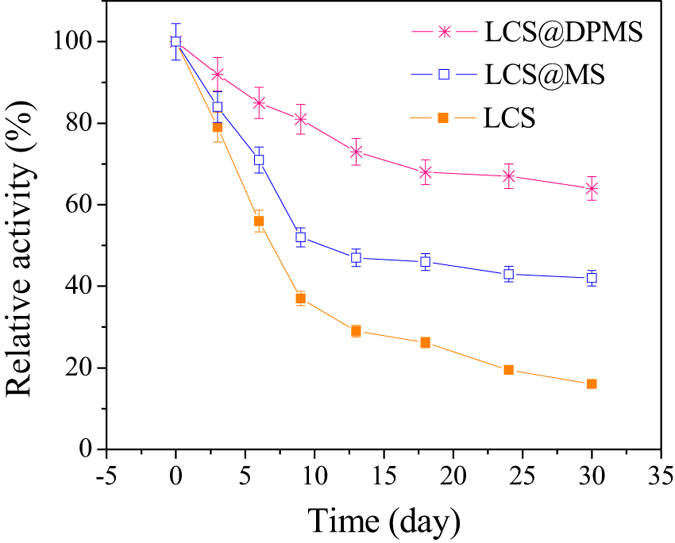
The storage stability of free and immobilized lipase. (The initial activity was 13.7 U/mg, 6.30 U/mg, and 7.26 U/mg for free lipase, LCS@MS, and LCS@DPMS, respectively).

The results of storage stability of the free and immobilized lipase are shown in Fig. 9. Evidently, the activity of the immobilized lipase decreased slowly than that of the free lipase. After 30 days, the relative activity of the LCS@DPMS could maintain 64%, whereas the relative activities of the free lipase and LCS@MS remained 16% and 42%, respectively. Thus, the LCS@DPMS exhibited higher storage stability than the free lipase and LCS@MS. The lipase was adsorbed in Meso-Onion-S via relatively weak physical force, and in contrast, the polydopamine on the surface of DPMS could react with the lipase molecules to form covalent conjugation, which could increase the conformational stability of lipase^34^,^35^. Therefore, the reason for the improved storage stability of LCS@DPMS might be that the LCS@DPMS could protect the active sites from deactivating more effectively under the same conditions compared with the free lipase and LCS@MS.

### The stability of free and immobilized lipase in shaking conditions

**Figure 10:**
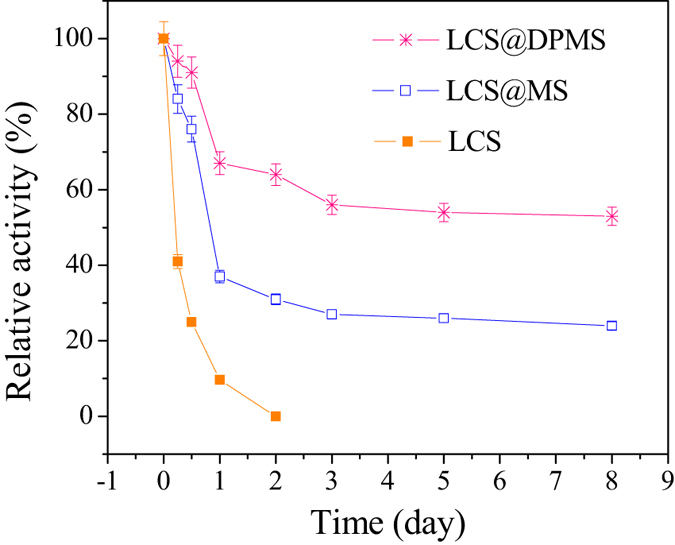
The stability of free and immobilized lipase in shaking conditions. (The initial activity was 13.7 U/mg, 6.30 U/mg, and 7.26 U/mg for free lipase, LCS@MS, and LCS@DPMS, respectively).

The stability of the immobilized enzyme in shaking conditions is one of the important issues for most enzymatic reactions that are conducted in vigorous shaking for reducing mass transfer limitations^2^. Figure 10. shows the stability of free and immobilized lipase in shaking conditions (200 rev/min). It could be seen that the activity of the free lipase decreased fast and only 9.7% of the initial activity was retained the next day, and the reason for the decrease of activity was that the vigorous shaking could result in the autolysis and denaturation of the lipase^9^,^36^. Adsorption of the enzyme in Meso-Onion-S and DPMS improved the stability of lipase, and the LCS@MS retained 24% of its initial activity after shaking for 7 days, and in contrast, the LCS@DPMS retained 53% of its initial activity under the same conditions. The activity reduction was ascribed to the leaching of enzyme from the immobilized lipase^37^,^38^. Compared to the LCS@MS, the improved storage stability of LCS@DPMS might be ascribed to the strong conjugation between the lipase molecules and the polydopamine on the surface of Meso-Onion-S, which could prevent the lipase leaching effectively.

### Kinetics of free and immobilized lipase

The kinetic parameters of the free lipase, LCS@MS and LCS@DPMS are listed in Table 1. The K_m_ values of the LCS@MS and LCS@DPMS were 1.76 and 1.58 times higher than that of the free lipase, respectively, indicating that the substrate’s affinity capacity for the immobilized lipase was lower compared to the free lipase. The change in the substrate’s affinity for the lipase was probably caused by conformational change in the adsorption process of lipase onto supports, and resulting in a lower accessibility of the substrate toward the active sites of the immobilized lipase^9^,^39^. Furthermore, the K_m_ value of the LCS@DPMS was lower than that of the LCS@MS, which might be ascribed to that the polydopamine on the surface of Meso-Onion-S could keep the conformation of the immobilized lipase more integrated and then enhance the affinity capacity between the lipase and the substrate^7^.

### Reusability of the immobilised lipases

**Figure 11:**
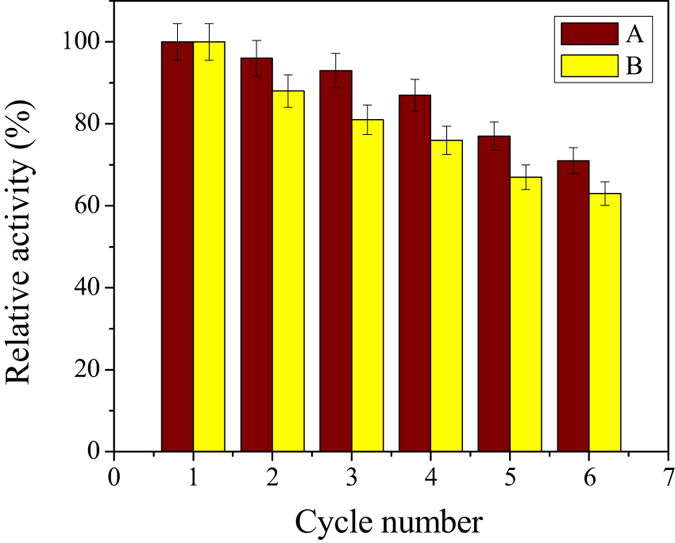
Reusability of (A) LCS@DPMS and (B) LCS@MS. (The initial activity was 13.7 U/mg, 6.30 U/mg, and 7.26 U/mg for free lipase, LCS@MS, and LCS@DPMS, respectively).

For practical applications, the reuse of immobilised lipase is necessary. The reusability of lipase immobilized in Meso-Onion-S and DPMS is shown in Fig. 11. It could be found that the LCS@MS and LCS@DPMS retained 63 and 71% of their initial activities after 6 consecutive operations, respectively. The decrease in activity was mainly caused by the denaturation and leaching of enzyme from the immobilised lipases^35^,^40^. Contrasting to LCS@MS, the activity of LCS@DPMS decreased slowly. The low operating stability of LCS@MS might result from that the lipase was adsorbed on Meso-Onion-S through physical force which was relatively weak and the lipase was easy to leach during the catalytic process^34^. The improved operational stability of LCS@DPMS was caused by the strong conjugation of covalent bond between the lipase molecules and the dopamine functionalized Meso-Onion-S, and thus the leakage of lipase from the DPMS was prevented. Moreover, the covalent bond could prevent the denaturation of lipase molecules upon use^31^,^32^,^33^. The more stable performance of LCS@DPMS in repeated use will make it desirable for practical applications.

## Conclusions

In summary, a simple and cost-effective method was used to immobilize lipase *Candida* sp. 99-125 in mesoporous onion-like silica based on grafting polydopamine onto the silica, and the pH, thermal and storage stability of the immobilized lipase LCS@DPMS were improved significantly. Additionally, the pH profile of LCS@DPMS was wider range than free lipase and LCS@MS, and the performance of its reusability was more stable than LCS@MS. Taken together, the DPMS had great potential as a matrix for practical applications in the immobilization of lipase.

## Additional Information

**How to cite this article**: Gao, J. *et al*. Dopamine-functionalized mesoporous onion-like silica as a new matrix for immobilization of lipase *Candida* sp. 99-125. *Sci. Rep.* **7**, 40395; doi: 10.1038/srep40395 (2017).

**Publisher's note:** Springer Nature remains neutral with regard to jurisdictional claims in published maps and institutional affiliations.

## Figures and Tables

**Table 1:** Kinetic parameters of free and immobilised lipases.

Samples	Free LCS	_LCS@MS_	_LCS@DPMS_
*K*_*m*_ (mM)	1.37 ± 0.06	2.41 ± 0.09	2.26 ± 0.08
*V*_*max*_ (mM/min)	89.2 ± 3.3	53.4 ± 1.39	61.6 ± 1.7

## References

[b1] Hartmann M. & Kostrov X. Immobilization of enzymes on porous silicas–benefits and challenges. Chem. Soc. Rev. 42, 6277–6289 (2013). 23765193 10.1039/c3cs60021a

[b2] Gao J., Shi L., Jiang Y., Zhou L. & He Y. Formation of lipase Candida sp. 99-125 CLEAs in mesoporous silica: characterization and catalytic properties. Catal. Sci. Technol. 3, 3353 (2013).

[b3] Terentyeva T. G. . Bioactive flake–shell capsules: soft silica nanoparticles for efficient enzyme immobilization. J. Mater. Chem. B 1, 3248 (2013). 10.1039/c3tb20461h 32261033

[b4] Ling D. . A general strategy for site-directed enzyme immobilization by using NiO nanoparticle decorated mesoporous silica. Chem. Eur. J. 20, 1–7 (2014). 10.1002/chem.201403071 24861357

[b5] Datta S., Christena L. R. & Rajaram Y. R. S. Enzyme immobilization: an overview on techniques and support materials. 3 Biotech. 3, 1–9 (2012). 10.1007/s13205-012-0071-7 PMC3563746 28324347

[b6] Hwang E. T. . Immobilization and stabilization of subtilisin Carlsberg in magnetically-separable mesoporous silica for transesterification in an organic solvent. Green Chem. 14, 1884 (2012).

[b7] Bai Y. X., Li Y. F., Yang Y. & Yi L. X. Covalent immobilization of triacylglycerol lipase onto functionalized novel mesoporous silica supports. J. biotechnol. 125, 574–582 (2006). 16697482 10.1016/j.jbiotec.2006.04.003

[b8] Jun S.-H. . Highly efficient enzyme immobilization and stabilization within meso-structured onion-like silica for biodiesel production. Chem. of Mater. 24, 924–929 (2012).

[b9] Gao J. . Formation of Nitrile Hydratase cross-linked enzyme aggregates in mesoporous onion-like silica: preparation and catalytic properties. Ind. Eng. Chem. Res. 54, 83–90 (2015).

[b10] Kim M. I. . Immobilization of Mucor javanicus lipase on effectively functionalized silica nanoparticles. J. Mol. Catal. B: Enzym. 39, 62–68 (2006).

[b11] Babaki M., Yousefi M., Habibi Z., Brask J. & Mohammadi M. Preparation of highly reusable biocatalysts by immobilization of lipases on epoxy-functionalized silica for production of biodiesel from canola oil. Biochem. Eng. J. 101, 23–31 (2015).

[b12] Jiang Y. . Facile immobilization of enzyme on three dimensionally ordered macroporous silica via a biomimetic coating. New J. Chem. 39, 978–984 (2015).

[b13] Lu S., An Z., He J. & Li B. Hierarchically-structured immobilized enzyme displaying the multi-functions of bio-membranes. J. Mater. Chem. 22, 3882 (2012).

[b14] Lee H., Dellatore S. M., Miller W. M. & Messersmith P. B. Mussel-inspired surface chemistry for multifunctional coatings. Science 318, 426–430 (2007). 17947576 10.1126/science.1147241 PMC2601629

[b15] Lee H., Rho J. & Messersmith P. B. Facile Conjugation of Biomolecules onto Surfaces via Mussel Adhesive Protein Inspired Coatings. Adv. Mater. 21, 431–434 (2009). 19802352 10.1002/adma.200801222 PMC2755254

[b16] Ren Y. . Facile, high efficiency immobilization of lipase enzyme on magnetic iron oxide nanoparticles via a biomimetic coating. BMC Biotechnol., 11, 63 (2011). 21649934 10.1186/1472-6750-11-63 PMC3212977

[b17] Sureshkumar M. & Lee C.-K. Polydopamine coated magnetic-chitin (MCT) particles as a new matrix for enzyme immobilization. Carbohyd. Polym. 84, 775–780 (2011).

[b18] de Souza R. L. . Protic ionic liquid as additive on lipase immobilization using silica sol-gel. Enzyme microb. tech. 52, 141–150 (2013). 10.1016/j.enzmictec.2012.12.007 23410924

[b19] Kharrat N., Ali Y. B., Marzouk S., Gargouri Y.-T. & Karra-Châabouni M. Immobilization of Rhizopus oryzae lipase on silica aerogels by adsorption: comparison with the free enzyme. Process Biochem. 46, 1083–1089 (2011).

[b20] Kalantari M., Kazemeini M., Tabandeh F. & Arpanaei A. Lipase immobilisation on magnetic silica nanocomposite particles: effects of the silica structure on properties of the immobilised enzyme. J. Mater. Chem. 22, 8385 (2012).

[b21] Izrael Živković L. T. . Immobilization of Candida rugosa lipase by adsorption onto biosafe meso/macroporous silica and zirconia. Biochem. Eng. J. 93, 73–83 (2015).

[b22] Lu J., Nie K., Wang F. & Tan T. Immobilized lipase Candida sp. 99-125 catalyzed methanolysis of glycerol trioleate: solvent effect. Bioresource technol. 99, 6070–6074 (2008). 10.1016/j.biortech.2007.12.045 18255281

[b23] Gao J. K., Hou L. A., Zhang G. H. & Gu P. Facile functionalized of SBA-15 via a biomimetic coating and its application in efficient removal of uranium ions from aqueous solution. J. Hazard. Mater. 286, 325–333 (2015). 25590826 10.1016/j.jhazmat.2014.12.061

[b24] Chen Y., Gao J., Wen X. & Wu W. Efficient removal of cadmium using facile functionalized of mesoporous silica via a biomimetic coating. RSC Adv. 6, 18340–18347 (2016).

[b25] Lee H., Scherer N. F. & Messersmith P. B. Single-molecule mechanics of mussel adhesion. P. Natl. Acad. Sci. USA 103, 12999–13003 (2006). 10.1073/pnas.0605552103 PMC1559742 16920796

[b26] Wu J. C. . Enhanced enantioselectivity of immobilized Candida antarctica lipase for hydrolysis of ketoprofen ethyl ester at pH 1. Korean J. Chem. Eng. 24, 648–650 (2007).

[b27] Joyce P., Whitby C. P. & Prestidge C. A. Interfacial processes that modulate the kinetics of lipase-mediated catalysis using porous silica host particles. RSC Adv. 6, 43802–43813 (2016).

[b28] Joyce P., Kempson I. & Prestidge C. A. Orientating lipase molecules through surface chemical control for enhanced activity: a QCM-D and ToF-SIMS investigation. Colloid. surface. B 142, 173–181 (2016). 10.1016/j.colsurfb.2016.02.059 26954083

[b29] Reis P. . Lipase-catalyzed reactions at different surfaces. Langmuir 22, 8169–8177 (2006). 16952258 10.1021/la060913s

[b30] Reis P., Holmberg K., Watzke H., Leser M. E. & Miller R. Lipases at interfaces: a review. Adv. colloid interfac. 147–148, 237–250 (2009). 10.1016/j.cis.2008.06.001 18691682

[b31] Yong Y. . Characterization of Candida rugosa lipase immobilized onto magnetic microspheres with hydrophilicity. Process Biochem. 43, 1179–1185 (2008).

[b32] Iyer P. V. & Ananthanarayan L. Enzyme stability and stabilization—Aqueous and non-aqueous environment. Process Biochem. 43, 1019–1032 (2008).

[b33] Pizarro C. . Influence of different immobilization techniques for Candida cylindracea lipase on its stability and fish oil hydrolysis. J. Mol. Catal. B: Enzym. 78, 111–118 (2012).

[b34] Wang H., Jiang Y., Zhou L., He Y. & Gao J. Immobilization of penicillin G acylase on macrocellular heterogeneous silica-based monoliths. J. Mol. Catal. B: Enzym. 96, 1–5 (2013).

[b35] Chen L., Wei B., Zhang X. & Li C. Bifunctional graphene/gamma-Fe(2)O(3) hybrid aerogels with double nanocrystalline networks for enzyme immobilization. Small 9, 2331–2340 (2013). 23423944 10.1002/smll.201202923

[b36] Lee J. . Magnetically-separable and highly-stable enzyme system based on crosslinked enzyme aggregates shipped in magnetite-coated mesoporous silica. J. Mater. Chem. 19, 7864 (2009).

[b37] Kim M. I. . Crosslinked enzyme aggregates in hierarchically-ordered mesoporous silica: a simple and effective method for enzyme stabilization. Biotechnol. Bioeng. 96, 210–218 (2007). 16986168 10.1002/bit.21107

[b38] Lee J. . Simple synthesis of hierarchically ordered mesocellular mesoporous silica materials hosting crosslinked enzyme aggregates. Small 1, 744–753 (2005). 17193518 10.1002/smll.200500035

[b39] Li Y. . Pore size of macroporous polystyrene microspheres affects lipase immobilization. J. Mol. Catal. B: Enzym. 66, 182–189 (2010).

[b40] Yiu H. H. P., Wright P. A. & Botting N. P. Enzyme immobilisation using SBA-15 mesoporous molecular sieves with functionalised surfaces. J. Mol. Catal. B: Enzym. 15, 81–92 (2001).

